# A dataset dedicated to the training of large- language models for agronomic management practices and production in Norwegian agriculture

**DOI:** 10.1016/j.dib.2025.111326

**Published:** 2025-01-23

**Authors:** Olena Bugaiova, Kristian Nikolai Jæger Hansen

**Affiliations:** Aarhus University, Department of Agroecology, Section for Systems Analysis and Sustainability, 8830 Tjele, Denmark

**Keywords:** Farming, Machine learning, Text data, Domain adaption

## Abstract

This dataset focuses on the agricultural management practices and production in Norway, derived from the websites Nibio.no, Plantevernleksikonet.no, and nlr.no. All gathered data is in Norwegian. The data is in JSON files (RAW format) and covers topics pertinent to Norwegian agriculture, such as crop rotation, soil health, plant protection and sustainable farming techniques. The data was collected by three Python scripts specially adapted to each website. The cleaned text data is valuable for training or evaluating Natural Language Processing (NLP) Models in an experimental context in Norway or adapting Large-Language Models (LLM) to the domain of Norwegian agriculture within the Norwegian language.

Specifications Table

**Table:** 

Subject	Data Science: Data Mining, Machine Learning, Statistics and Agricultural Science.
Specific subject area	*Agronomic Management and Production, NLP and LLM.*
Type of data	RAW, Web data; Text data.
Data collection	Using custom-developed Python pipelines in GoogleCollab.
Data source location	List of all websites: nibio.no, plantevernleksikonet.no, nlr.no.
Data accessibility	From the following repositories Repository name: Kaggle, Github Data identification number: Direct URL to data: https://doi.org/10.34740/KAGGLE/DSV/9037685 and https://doi.org/10.5281/zenodo.13371101
Related research article	*none.*

## 1 Value of the Data

• The dataset contributes to the resources available for NLP in specialised areas, mainly focusing on Norwegian agricultural management practices and production within the Norwegian language.
• The dataset is beneficial for fine-tuning or Retrieval-Augmented Generation (RAG) of existing LLMs within an agronomic context.
• The dataset can also be used for various analytical tasks, such as exploring common
• advisory and agricultural practices in Norway.
• The dataset is designed to be scalable, with all processing codes included, allowing for easy replication and expansion with additional websites using the Python code.

## 2 Background

Agricultural practices vary widely across regions and countries due to differences in management techniques, local climate, pathogens, weeds and environmental factors. These variations affect how the optimal management looks in different regions, for example, as the prevalence of pathogens and weeds differs, thus influencing the need for production alterations to obtain a high yield. Farmers often rely on extension services to guide pest and weed management, but also for fertiliser strategies and improved environmental sustainability. LLMs, therefore, are likely to have a high potential within an agronomic context, however, they need to be updated and retrained with the latest regional information and within the national language applied to provide relevant information giving agronomic advice. Lack of location-specific knowledge has earlier been shown to be a limitation of the capability of LLMs to provide valuable answers to agronomic problems [1].

As LLMs, and AI generally, have changed the global technological landscape, many communities across the world have been left unsupported due to the language limitations of existing models [2] Training of LLMs requires substantial amounts of text data, which in most languages other than English is not readily available. <0.1 % of the text on webpages exists in Norwegian [3], and according to some literature, Norwegian can be described as a low-resource language [[4], [5]]. This gap hinders the applicability and usefulness of generative AI, and it has the potential to further widen existing disparities.

In principle, there are two main approaches to adapting LLMs: RAG vs Fine-tuning (e.g., [1]). LLMs are likely to have a high potential within an agronomic context, while, as earlier discussed, the application is limited due to a lack of specific data [1]. In addition, there is currently a lack of easily accessible text data (i.e., retrieved data and readily available through general search engines) for adapting LLMs for agriculture in the Norwegian language. For example, Google Dataset Search which helps researchers locate online data that is freely available for use shows that there are 42 datasets found for Norwegian" AND “Agriculture”, 100+ datasets found for Norwegian" AND “Text” and 100+ datasets found for “Agriculture" AND “Text”. But there are no datasets for “text” AND “Agriculture” AND “Norwegian”. In comparison, there are 13 datasets for the combination of “text” AND “Agriculture” AND “English” for the time when this research paper is written. One such dataset is Ergonomics/Human Factors in agricultural sectors [6].

This text data collection aims to construct a dataset describing typical management factors in Norwegian agricultural production. It addresses two main challenges when deploying LLMs to new domains: language adaptation and domain-specific knowledge. The dataset size is comparable to the one used in another similar project, like the project for the Ukrainian language from Grammarly [7]. The dataset can be used to improve pre-trained LLMs (fine-tuning or RAG) or implemented into existing datasets in the Norwegian language. Our goal is to facilitate the creation of LLMs better adapted to the use cases in Norwegian language.

## 3 Data Description

The data collected resulted in 2292 articles in the combined dataset (Table 1). The data gathered about agriculture was all in Norwegian and only contained agricultural information. Collected text data was manually validated on a random subset of 20 articles for consistency. After clean-up of the vocabulary by removing typical stop words in Norwegian, we made visualization to give a better sense of data; see Agriculture_Text_Visualization for further description.

**Table 1:** Quantitative descriptive statistics of the dataset collected.

File name	Website	Number of articles	Number of sentences in the text	Number of tokens in the text	Size of the text vocabulary
nibio_text_data.json	nibio.no	497	6970	144,511	16,330
plantevernleksikonet_te xt_data.json	plantevernleksikonet.no	1090	33,928	525,705	27,092
nlr_text_data.json	nlr.no	707	28,099	508,016	35,127
					
text_on_agriculture.json	Combined	2294	71,147	1,190,123	61,906

The collected text data comprises two components: 1) one folder with the gathered text data in four different files: three for preprocessed data (one for each web page), and one for all webpages, and 2) one folder with three different phyton scripts for collecting the data, and one for data cleaning, visualization, and transformation. Combined text data is structured as the title, and article text. Data is represented as raw text (without tokenization and not specifically fine-grained for the agricultural language). The dataset and files are structured to facilitate the easy inclusion of new information and are adaptable through modifications to the Python script for new websites. We stored all the text data in JSON format files described under the Data Explorer section.

The procedure for collecting the web data for each article was as followed: under each header, we retrieve paragraphs. Then, we combine paragraphs into text separated by a new line symbol. When we retrieve list items, we combine them into text separated by commas. Then, we combine text retrieved from three websites into one and remove repeated punctuation symbols, i.e. repeated new line symbols or dots. Then, the text was stored in a JSON format.

**Fig. 1:**
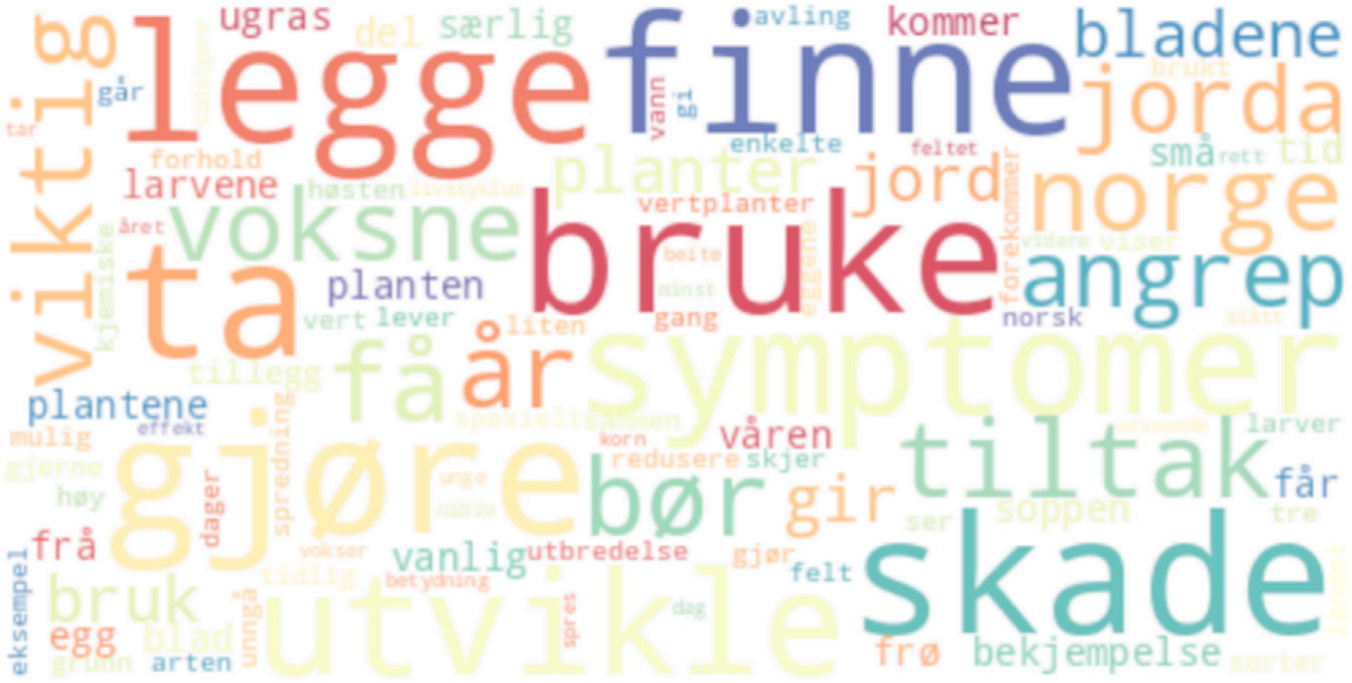
Word cloud of the data. The word cloud was constructed by removing the dataset's most common stop and conjunction words.

**Fig. 2:**
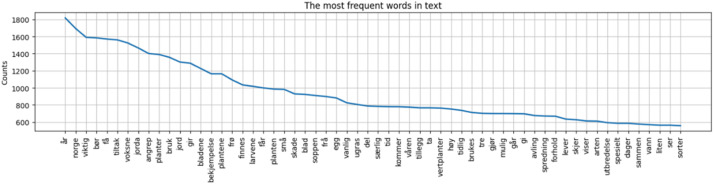
Most frequent words in the text.

To describe the data quantitatively and with visualizations we used sent_tokenize and word_tokenize methods from NLTK tool v3.2.4. The tokenization was used to describe the data quantitatively and with visualizations. To calculate the vocabulary size, we retrieve a unique set of tokens from the text and clean the tokens by removing stop words, punctuation, special symbols, words consisting of only one symbol, numbers and arithmetic expressions. Table 2 and 3 Figs. 1 and 2

**Table 2:** Data files and location.

File Name	Description	Location of File
nibio_text_data.json	text from nibio.no	text/nibio_text_data.json
plantevernleksikonet_text_data.json	text from plantevernleksikonet.no	text/plantevernleksikonet_text_data.json
nlr_text_data.json^1^	text from nlr.no	text/nlr_text_data.json
text_on_agriculture.json	text combined	text/text_on_agriculture.json
Agriculture_Text_Visualization.ipynb	Python code for data transformation, validation, and visualization	code/Agriculture_Text_Visualization.ipynb
NIBIO_Web_Scraping_of_Agriculture_Text.ipynb	Python code to retrieve data from nibio.no	code/NIBIO_Web_Scraping_of_Agriculture_Text.ipynb
NLR_Web_Scraping_of_Agriculture_Text.ipynb	Python code to retrieve data from plantevernleksikonet.no	code/NLR_Web_Scraping_of_Agriculture_Text.ipynb
Plantevernleksikonet_Web_Scraping_of_Agriculture_Text.ipynb	Python code to retrieve data from nlr.no	code/Plantevernleksikonet_Web_Scraping_of_Agriculture_Text.ipynb

1 The data from NLR can be expanded in the future, gathering more text data.

**Table 3:** Rank of words, bigrams and trigrams in the total dataset.

Rank	Words	Bigrams	Trigrams
1	år	formeringen spredningen	formeringen spredningen skjer
2	Norge	opptrer ugras	voksne planten høy
3	viktig	overlevelse spredning	biologi formeringen spredningen
4	bør	sterke angrep	skade/ulempe opptrer ugras
5	få	spredningen skjer	kjennetegn voksne planten
6	tiltak	planten høy	hører biologiske gruppen
7	voksne	voksne planten	opptrer ugras hager
8	jorda	biologiske gruppen	spredningen skjer utelukkende
9	angrep	organisk materiale	opptrer ugras beite
10	planter	forebyggende tiltak	opptrer ugras slags
11	bruk	kjemiske midler	bekjempelse forebyggende tiltak
12	jorda	legger egg	jord opptrer ugras
13	gir	biologi formeringen	spredningen skjer frø
14	bladene	betydningen vokseplasser	opptrer ugras åkerkulturer
15	bekjempelse	stengelen opprett	opptrer ugras åker

### 3.1 Data Visualization

## 4 Experimental Design, Materials and Methods

Text data was collected from three Norwegian agricultural-related websites. The information gathered from the websites includes data from the largest advisory service for the agricultural sector, Norsk landbruksrådgivning (Norwegian Agricultural Extension Service, NLR), the most prominent agricultural research institute in Norway, Norsk Institutt for Bioøkonomi (Norwegian Institute for Bioeconomy, NIBIO), and the most comprehensive web page dedicated to plant protection in agriculture, Plantevernleksikonet also provided by NIBIO. These websites were selected since they are based upon earlier conducted scientific work, revised and edited by agronomists, thus being a high-quality data source. The text data was collected on 11.07.2024 from all three websites. The dataset was constructed by extracting all information from the websites using Python, BeautifulSoup, and NLTK for tokenization when describing the data.

## Limitations

Not applicable.

## Ethics Statement

The authors have read and followed the ethical requirements for publication in Data in Brief and confirm that the current work does not involve human subjects, animal experiments, or any data collected from social media platforms. The authors declare that the work described in this paper is original and not under consideration for publication elsewhere, in whole or in part. Its publication is approved by all the authors listed.

## Credit Author Statement

**Olena Bugaiova:** Data collection, Validation, Conceptualization, Methodology, Investigation, Data curation and Writing – review & editing. **Kristian Nikolai Jæger Hansen:** Conceptualization, Writing – original draft and Supervision.

## Data Availability

Githubcollecting-data-about-norwegian-agriculture (Original data). KaggleAbout Norwegian Agriculture (Original data).
